# The association between multimorbidity patterns and physical frailty among middle-aged and older community-dwelling adults: the mediating role of depressive symptoms

**DOI:** 10.3389/fpubh.2025.1527982

**Published:** 2025-05-01

**Authors:** Yuhan Geng, Ming Zhou, Yangxiaoxue Liu, Tianshu Zhao, Jiali Zhang, Min Xin, Wenxin Wang, Gongzi Zhang, Liping Huang

**Affiliations:** ^1^Medical School of Chinese PLA, Beijing, China; ^2^Department of Rehabilitation Medicine, The First Medical Center of Chinese PLA General Hospital, Beijing, China

**Keywords:** multimorbidity, frailty, depressive symptoms, cluster analysis, mediation analysis

## Abstract

**Background:**

This study aimed to investigate the association between multimorbidity and frailty, and the potential mediating role of depressive symptoms in Chinese middle-aged and older community-dwelling adults.

**Methods:**

We selected a total of 5,232 adults with two or more chronic diseases from the China Health and Retirement Longitudinal Study (CHARLS) database. Clusters of participants with similar multimorbidity patterns were identified through fuzzy c-means cluster analyses. The cross-sectional association between multimorbidity and frailty was measured through logistic regression analyses. Mediation analysis was applied to examine direct and indirect associations within the counterfactual framework.

**Results:**

At baseline, we identified five multimorbidity patterns. Two of these patterns significantly increased the risk of frailty compared to a non-specific pattern. Depression mediated 35.20% of the effect of multimorbidity on frailty (*p* = 0.042). Notably, in adults aged 60 years and older, this mediation accounted for 69.84% of the total effect, surpassing the direct impact of multimorbidity on frailty. Among individuals with economic support (0.020, 95% CI: 0.002–0.040), high school education (0.062, 95% CI: 0.007–0.120), and no alcohol consumption (0.024, 95% CI: 0.003–0.050), depression entirely mediated the impact of comorbidities.

**Conclusion:**

This study reveals strong links between specific multimorbidity patterns and physical frailty, with depression significantly mediating these effects, particularly in certain populations. Findings emphasize tailored mental health interventions’ necessity in specific groups.

## Introduction

Frailty is a clinical syndrome characterized by diminished physiological reserve and heightened vulnerability to adverse health outcomes, typically defined as meeting ≥3 of the Fried phenotypic criteria (unintentional weight loss, weakness, self-reported exhaustion, slow gait speed, and low physical activity); pre-frailty refers to the presence of 1–2 criteria and represents a transitional state toward full frailty (1, 2). This decline in multisystem resilience compromises the body’s ability to cope with stressors, accelerating functional deterioration (1). China’s accelerated population aging, projected to have 366 million adults ≥65 by 2050, creates unique urgency for frailty research (3). As the global population ages, the impact of frailty is expected to increase, especially in low- and middle-income countries, where people from lower socioeconomic backgrounds and ethnic minorities tend to have higher rates of frailty (4). China’s rural frailty prevalence (consistently above 20%) markedly exceeds urban rates (14.3%) and Western averages (10–15%) (5, 6), reflecting distinct challenges in healthcare access. Approximately 25% of older adults in Asia are diagnosed with frailty (7), and its consequences—including disability, falls, fractures, mobility loss, loneliness, reduced quality of life, depression, cognitive decline, dementia, hospitalization, and institutionalization—impose significant burdens on individuals and healthcare systems (8). Traditional family care structures are weakening alongside rapid urbanization, while institutional support remains limited (9). In the United States, aging, frailty, and chronic diseases account for over 80% of healthcare expenditures, with costs doubling for those with mobility impairments (10). China’s dual burden of chronic diseases and constrained health resources underscores the need for cost-effective prevention strategies. Identifying modifiable risk factors for frailty is thus essential, particularly in China where environmental exposures, nutritional disparities, and sociocultural transitions interact uniquely with aging processes (11–13).

Multimorbidity, which refers to having two or more chronic diseases at the same time, is not merely associated with frailty but actively drives its progression through interconnected biological and social pathways (14). Chronic disease clusters (e.g., cardiometabolic and inflammatory conditions) induce sustained systemic inflammation (elevated IL-6, CRP) and metabolic dysregulation, which directly impair muscle synthesis and stress adaptation capacity—core features of frailty (15, 16). The prevalence of multimorbidity increases significantly with age, particularly after the age of 45, where more than 30% of older adult Chinese adults are affected. After the age of 60, more than 35% of older adult Chinese adults are affected (7). Moreover, nearly one-fifth of middle-aged individuals (aged 45–59 years) also have multimorbidity (17). Critically, the social consequences of managing multiple diseases—such as fragmented healthcare access in rural China and financial strain from out-of-pocket costs—further exacerbate frailty by limiting rehabilitation opportunities and promoting physical inactivity (18). A systematic review found that 16% of individuals with ≥2 chronic conditions meet frailty criteria (10), and both frailty and pre-frailty prevalence escalate alongside multimorbidity (19). Frail individuals with multimorbidity face markedly elevated risks of adverse outcomes, including 4.2-fold higher mortality, 3.3-fold increased likelihood of prolonged hospitalization, and 1.8-fold greater risk of 30-day unplanned readmissions compared to non-frail counterparts (20).

However, the relationship between frailty and multimorbidity often produces inconsistent findings due to limitations such as small sample sizes in many studies. While some studies suggest a weak association (10), others indicate that the number of chronic conditions accelerates frailty progression (21). Critically, multimorbidity often manifests as disease clusters (e.g., metabolic syndrome, chronic inflammation) (22), and merely counting diseases fails to capture this complexity (23). Identifying multimorbidity patterns—rather than isolated conditions—has emerged as a superior approach to understanding frailty risk (24). For instance, clusters involving metabolic and inflammatory diseases are stronger predictors of frailty than disease counts alone (25), underscoring the need to analyze disease interactions.

Traditionally, frailty assessment has predominantly focused on declines in physical function and mobility (26). However, emerging evidence from recent studies and clinical practice suggests that frail individuals may also experience declines in cardiopulmonary function and brain function (27, 28). To explore this, our study focuses on how multimorbidity may contribute to frailty by mediating the decline in brain function, with depression identified as a particularly important candidate. Beyond direct associations, emerging research highlights mediators that may explain how multimorbidity exacerbates frailty. Systemic inflammation, driven by chronic diseases, is one plausible pathway (25). For example, inflammatory markers like C-reactive protein and interleukin-6 are elevated in both multimorbidity and frailty, potentially accelerating muscle catabolism and functional decline (25). Psychosocial factors, such as depression and social isolation, are additional candidates (29). Depression is prevalent in multimorbid populations—72% of older adults with ≥3 chronic conditions in northern Italy reported depressive symptoms (30)—and is independently associated with frailty (31). Emerging evidence indicates that targeting depression in this population can yield clinical benefits: a randomized controlled trial among multimorbid older adults found that cognitive behavioral therapy (CBT) reduced depressive symptoms by 40% and delayed frailty progression by 6 months compared to usual care (32). Social isolation may compound these effects by reducing access to care and physical activity (33). These findings underscore the critical role of identifying modifiable mediators, as targeting inflammation or psychosocial factors could provide novel interventions to delay frailty progression. However, mechanistic studies linking multimorbidity-frailty pathways remain scarce, with limited understanding of how biological (e.g., inflammation), psychological (e.g., depression), and social (e.g., isolation) mediators interact and contribute to frailty outcomes (34). Future research is needed to disentangle these complex relationships and develop precision interventions for vulnerable populations.

This study examines how multimorbidity drives frailty development, focusing on inflammatory and psychosocial mediators. Using CHARLS cross-sectional data, we identify multimorbidity clusters, assess their frailty associations, and quantify depressive symptoms’ mediating role. While causality requires longitudinal validation, our disease cluster approach aligns with systemic health burden frameworks (35), providing groundwork to prioritize high-risk groups (e.g., cardiometabolic clusters) for targeted interventions. Findings support integrating strength training (countering inflammation) and social activities (mitigating psychosocial risks) into frailty prevention policies (36). For instance, targeted strength training programs could counteract the inflammatory pathways linking multimorbidity to frailty, while group-based physical activities may address psychosocial mediators like depressive symptoms by enhancing social cohesion. This dual approach aligns with evidence that strength training not only prevents frailty and falls but also preserves functional autonomy (37), and that social physical activity interventions improve both mental health and community engagement in aging populations (38). Such strategies could be prioritized for at-risk groups identified through multimorbidity clustering (e.g., cardiometabolic or musculoskeletal clusters), thereby optimizing resource allocation in public health systems.

## Methods

### Study population and design

The study population was derived from a nationwide prospective cohort study, the China Health and Retirement Longitudinal Study (CHARLS), focusing on middle-aged individuals (aged≥45) (39). This study utilized a household survey to gather high-quality micro-data representing Chinese community-dwelling individuals, covering demographic characteristics, socioeconomic status, chronic diseases, physical function, healthcare, insurance, etc. CHARLS employs a multi-stage and random probability sampling method, with sampling conducted in 150 counties/districts across 28 provinces in China, ensuring good representativeness (24). All participants provided voluntary informed consent. The procedures for this survey were approved by the Institutional Review Board at Peking University (IRB00001052-11015).

To establish multimorbidity patterns, we excluded 10,664 individuals with fewer than two comorbid conditions, retaining only those with multiple comorbidities for subsequent analysis. We then excluded 583 participants who had missing data on key disease diagnoses, essential covariates, or frailty-related indicators to ensure the completeness of the data and maintain analytical rigor. Then, a total of 5,232 participants were included in this study (Supplementary Figure 1).

### Outcome: physical frailty assessment

Frailty was defined according to the Fried phenotype criteria (2), requiring ≥3 of the following five components. All measurements were standardized across the CHARLS cohort as follows:

1. Weakness: Weakness was defined according to Fried et al. (2) using sex- and BMI-specific cutoffs for maximal grip strength. Participants’ handgrip strength was measured three times on each hand using a dynamometer, with the highest value recorded for analysis. Thresholds were set as follows: for men, weakness was defined as ≤26 kg if BMI < 24 or ≤24 kg if BMI ≥ 24; for women, the corresponding thresholds were ≤18 kg (BMI < 24) or ≤16 kg (BMI ≥ 24) (2). Individuals unable to complete the test due to medical contraindications (e.g., severe arthritis, recent hand surgery) were categorically classified as “weak.”
2. Slowness: Slowness means being below the 20th sex-specific percentile. A Timed Up and Go (TUG) test was used to measure gait speed (40). The TUG test required research participants to get out of an armchair, walk three meters, and then get back in and sit down. The test started when the individual’s back left the armchair, and it ended when their buttocks made contact with the chair’s seat once more (41, 42).
3. Exhaustion: Exhaustion refers to the response to two items from the CES-D: (1) “I thought that everything I did was an effort”; and (2) “I could not get going.” If the participants feel tired all of the time, at least 3 or 4 days per week, they would be determined to be positive (2).
4. Weight loss: weight loss refers to the weight that has decreased by 5 kg or current body mass index (BMI) ≤ 18.5 kg/m2 during the last 12 months (43, 44).
5. Low activity: The physical activity questions in the Health Survey for England were taken from a validated physical activity interview (45). A question regarding the frequency of moderate activity (such as dancing, walking at a moderate pace, cleaning the car, gardening, floor, or stretching exercises) was answered by the participants. Low physical activity was defined as answering “hardly ever” or “never.” It was different from that proposed by Fried et al. (2). In Sun’s research, low physical activity has been evaluated using similar treatment variables (46).

### Chronic diseases assessment

Chronic diseases were gathered by self-reporting of the diagnoses. Participants were asked questions such as, ‘Have you been diagnosed with… by a doctor?’ The study focused on the 16 most common or important chronic diseases, as investigated in the CHARLS questionnaire, with participants providing binary responses indicating presence or absence (47). Validation studies in CHARLS indicate moderate agreement between self-reported hypertension and diabetes and biomedical measurements (*κ* = 0.57–0.65), with higher specificity (96.3–98.3%) than sensitivity (56.3–61.5%) (48). This suggests that while self-report may underdiagnose conditions, it is reliable for excluding unaffected individuals.

To ensure robust statistical analysis and avoid spurious findings, diseases with a prevalence of less than 2% were excluded (nervous or psychiatric problems, prostate diseases, and glaucoma). The chronic diseases included in the study are hypertension, dyslipidemia (elevated total cholesterol), diabetes or high blood sugar, cancer or malignant tumors (excluding mild skin cancers), chronic lung diseases (chronic bronchitis, emphysema, excluding tumors or cancer), liver disease (except fatty liver, tumors, and cancer), heart disease (coronary heart disease, angina, or congestive heart failure), stroke, kidney disease (excluding tumors or cancer), stomach or other digestive diseases (except for tumors or cancer), emotional, memory-related diseases (defined as diseases related to memory in this study, including dementia, brain atrophy, and Parkinson’s disease), and arthritis or rheumatism.

### Depression symptoms assessment

Depression was assessed using the Center for Epidemiologic Studies Depression Scale (CES-D) in the questionnaire (49), widely recognized as a measure of mental health status (50). The CES-D consists of 10 items with a total score of 30 points, with a score of 10 points or more indicating depression. Life satisfaction was categorized as ‘good,’ ‘fair,’ and ‘poor.’ Higher CES-D scores have earlier been shown to be associated with more severe depressive symptoms (51).

### Covariates

In the logistic regression analysis, we examined the association between multimorbidity patterns and frailty while adjusting for several covariates to account for potential confounding factors. The covariates included age (continuous), gender, education, financial support, marital status, history of drinking, history of smoking, place of residence, co-residence with children and number of family members (continuous). Gender was categorized as male or female. Education was classified as illiterate (never attended school), elementary and middle school (less than 9 years), high school (9–12 years), and university (13 years or more). Marital status was categorized as married with spouse present, separated, divorced, or unmarried (widowed or never married). Smoking history was categorized as ‘yes’ (former or current smoker) or ‘no’ (never smoked). Alcohol consumption was categorized as ‘yes’ (former or current drinker) or ‘no’ (never drank). Place of residence was classified as urban or rural. Average household income (Insurance and financial support) was categorized as ‘Yes’ or ‘No’. Co-residence with children was categorized as ‘Yes’ or ‘No’. These covariates were selected based on their established associations with both multimorbidity and frailty as reported in the existing literature.

### Statistical analyses

To identify patterns of multimorbidity, all participants with at least two chronic diseases were included. Dimensionality reduction was performed using multiple correspondence analysis, considering diseases with a prevalence >2%. Subsequently, a fuzzy c-means cluster algorithm was employed to generate clusters of individuals. Fuzzy c-means clustering is a soft clustering technique that allows each individual to have partial membership in multiple clusters, rather than being assigned to a single cluster exclusively. This approach is particularly suitable for analyzing multimorbidity patterns because it accommodates the overlapping and heterogeneous nature of chronic diseases. Utilizing a fuzzy clustering approach allowed for handling the stochastic nature of disease associations, potential noise from measurements (e.g., disease assessment), and variance due to between-individual differences more effectively. This method offers a deeper understanding of disease patterns by uncovering the probabilistic relationships between diseases and individuals. This approach allowed individuals to belong to more than one cluster, with each participant assigned a membership probability to each cluster. We then tested fuzziness parameter (m) values of 1.1, 1.2, 1.4, 1.5, 2, and 4 across a range of 1 to 20 clusters, and found that m = 1.1 performed the best, offering an optimal balance between cluster separation and membership probabilities. To evaluate clustering quality, we used validation indices, including the Fukuyama Index, Xie–Beni Index, Partition Coefficient Index and Partition Entropy Index. Given the stochastic nature of clustering, 100 independent repetitions were conducted to obtain the average final solution (52).

Participants were assigned to the cluster with the highest membership probability, while still acknowledging the possibility of shared diseases across clusters, illustrating the real-world complexity of multimorbidity. To characterize the clusters in terms of diseases, observed/expected ratios were calculated by comparing disease prevalence in the cluster with that of the overall sample population. Additionally, disease exclusivity was determined as the fraction of individuals with a particular disease in a cluster divided by the total number of individuals with that disease. Diseases were considered associated with specific clusters if the exclusivity was ≥25% or the observed/expected ratio was ≥2. Diseases meeting both criteria (exclusivity ≥25% and observed/expected ratio ≥2) were included in the cluster’s name (53). This dual-criterion approach ensures the identification of disease-cluster associations that are both statistically robust and clinically significant (54).

Logistic regression was employed to examine the cross-sectional association between multimorbidity patterns and baseline frailty. Models were initially adjusted for sociodemographic characteristics (age, sex, smoking and alcohol history), followed by additional adjustments for demographic factors (civil status and marital status), then followed by additional adjustments for co-residence with children and number of family members. This stepwise approach enabled us to assess the independent contribution of multimorbidity patterns to frailty while controlling for additional confounding variables at each stage.

## Results

### Prevalence of five multimorbidity patterns and related variables

A total of 5,232 individuals aged over 45 years and meeting the inclusion criteria participated in this study. The data cleaning process is depicted in Supplementary Figure S1. The mean age of participants in the cross-sectional analyses was 55.1 ± 10.8 years, with 53.1% being female. Five patterns of multimorbidity were identified at baseline: Cerebrovascular, respiratory, and hepatic diseases (*n* = 1,289, 24.6%); psychiatric and cerebrovascular diseases (*n* = 119, 2.3%); memory-related and metabolic disorders (*n* = 705, 13.5%); cancer (*n* = 102, 1.9%); and an unspecific pattern (*n* = 3,017, 57.7%) lacking over-represented diseases (see Supplementary Tables S1, S2 for details; Supplementary data are available). Table 1 presents the baseline characteristics of the total population (*n* = 5,232) according to the patterns of multimorbidity. Participants in the cancer pattern (55.9 ± 11.1 years), followed by the cardiovascular, respiratory & hepatic diseases pattern (54.9 ± 10.4 years), were the oldest. Those in the psychiatric, cardiovascular diseases pattern presented with the highest number of chronic diseases (3.5 ± 1.3). The unspecific pattern was relatively healthier, characterized by a younger average age (54.5 ± 10.5) and the lowest number of chronic diseases (2.4 ± 0.7).

**Table 1:** Sociodemographic characteristics and key variables of participants by multimorbidity pattern.

Characteristics	Unspecific (*n* = 3,017, 57.7%)	Cerebrovasc., Resp. & Hepatic (*n* = 1,289,24.6%)	Psychiatric & Cerebrovasc. (*n* = 119,2.3%)	Memory & Metabolic (*n* = 705,13.5%)	Cancer (*n* = 102,1.9%)	*p-*value	Total (n = 5,232)
Age	54.5 ± 10.5	54.9 ± 10.4	55.2 ± 11.9	54.8 ± 10.1	55.9 ± 11.1	0.481	55.06 ± 10.8
Sex (female)	1,618 (53.6)	648 (50.3)	61 (51.3)	394 (55.9)	55 (53.9)	0.143	2,776 (53.1)
Education							
Illiterate	633 (21)	250 (19.4)	20 (16.8)	162 (23)	25 (24.5)	0.896	1,090 (20.8)
Middle and primary school	1983 (65.7)	862 (66.9)	82 (68.9)	451 (64)	65 (63.7)		3,443 (65.8)
High school	339 (11.2)	147 (11.4)	15 (12.6)	79 (11.2)	10 (9.8)		590 (11.3)
University and above	62 (2.1)	30 (2.3)	2 (1.7)	13 (1.8)	2 (2)		109 (2.1)
Marital status							
Married	2,371 (78.6)	1,029 (79.8)	102 (85.7)	560 (79.4)	80 (78.4)	0.909	4,142 (79.2)
Separated	210 (7)	87 (6.7)	6 (5)	43 (6.1)	5 (4.9)		351 (6.7)
Divorced	31 (1)	14 (1.1)	1 (0.8)	9 (1.3)	0 (0)		55 (1.1)
Widowed	386 (12.8)	154 (11.9)	10 (8.4)	88 (12.5)	16 (15.7)		654 (12.5)
Never married	19 (0.6)	5 (0.4)	0 (0)	5 (0.7)	1 (1)		30 (0.6)
Residential area							
City	2,726 (90.4)	1,153 (89.4)	109 (91.6)	624 (88.5)	87 (85.3)	0.272	4,699 (89.8)
country	291 (9.6)	136 (10.6)	10 (8.4)	81 (11.5)	15 (14.7)		533 (10.2)
Average household income	200 (6.6)	90 (7)	5 (4.2)	55 (7.8)	5 (4.9)	0.528	355 (6.8)
Ever current alcohol	938 (31.1)	425 (33)	35 (29.4)	235 (33.3)	31 (30.4)	0.612	1,664 (31.8)
Ever current smoke	1,072 (35.5)	590 (45.8)	43 (36.1)	309 (43.8)	28 (27.5)	**<0.001**	2042 (39.0)**
Number of diseases	2.4 ± 0.7	3.3 ± 1.1	3.5 ± 1.3	3.1 ± 1.4	3.2 ± 1.5	**<0.001**	3.1 ± 1.2**
Frail	170 (5.6)	115 (8.9)	18 (15.1)	38 (5.4)	8 (7.8)	**<0.001**	349 (6.7)**

Cerebrovasc., Resp. & Hepatic = Cerebrovascular disease, Disease of Respiratory system & Hepatic disease; Psychiatric & Cerebrovasc. = Psychiatric & Cerebrovascular disease; Memory& Metabolic = Memory Related Disease & Metabolic. Values are presented as absolute number and column percentage (%) or mean ± standard deviation. ∗*p* < 0.01. ∗∗*p* < 0.001.

### The effect of multimorbidity exposure on physical frailty

**Figure 1:**
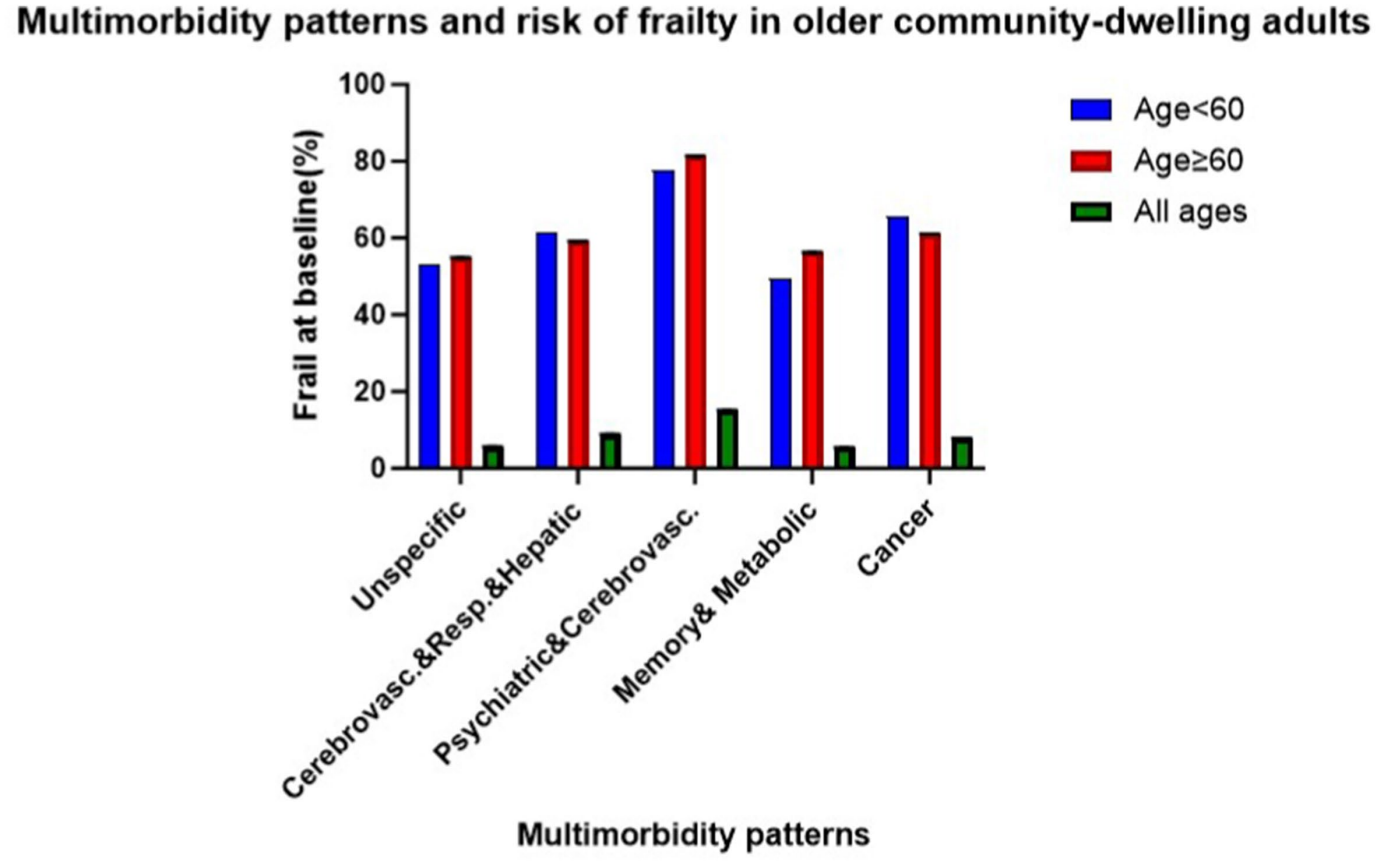
Baseline physical frailty prevalence by multimorbidity pattern.

Overall, 349 (7%) participants were identified with physical frailty at baseline, with the highest prevalence observed in the psychiatric, cardiovascular diseases pattern (15%) and the lowest in the memory-related, metabolic disorders pattern (5%). Across all age groups, prevalence was higher in older cohorts within the psychiatric, cardiovascular diseases pattern, memory-related, metabolic disorders pattern, and unspecific pattern (see Figure 1). In the fully adjusted cross-sectional analyses, two patterns showed statistically significant associations with frailty compared to the unspecific pattern. The odds ratios were 1.66 (95% CI: 1.30–2.13) for the cardiovascular, respiratory, and hepatic diseases pattern and 3.05 (95% CI: 1.80–5.16) for the psychiatric and cardiovascular diseases pattern (see Table 2).

**Table 2:** Cross-sectional association between multimorbidity patterns and physical frailty in complete cases at baseline (*n* = 5,232).

Pattern	Case		Model 1		Model 2		Model 3	
	n/N	%	OR	OR	OR	95%CI	OR	95%CI
Unspecific	170/3017	5.6	1(ref)	—	1(ref)	—	1(ref)	—
Cerebrovasc., Resp. & Hepatic	115/1289	8.9	1.66	1.29–2.12	1.64	1.30–2.13	1.64	1.28–2.10
Psychiatric & Cerebrovasc.	18/119	15.1	3.02	1.78–5.11	3.05	1.80–5.16	3.10	1.77–5.13
Memory & Metabolic	38/705	5.4	0.96	0.67–1.38	0.96	0.67–1.38	0.94	0.65–1.34
Cancer	8/102	7.8	1.41	0.67–2.96	1.42	0.68–2.97	1.44	0.63–2.84

Cerebrovasc., Resp. & Hepatic = Cerebrovascular disease, Disease of Respiratory system & Hepatic disease; Psychiatric & Cerebrovasc. = Psychiatric & Cerebrovascular disease; Memory& Metabolic = Memory Related Disease & Metabolic; OR = odds ratio; CI = confidence interval. Model 1: adjusted for age, sex, smoking status and alcohol consumption. Model 2: adjusted for age, sex, education, marital status, smoking status and alcohol consumption. Model 3: adjusted for age, sex, education, marital status, smoking status, alcohol consumption, co-residence with children and number of family members.

### Identification of the mediating effect of depression

**Figure 2:**
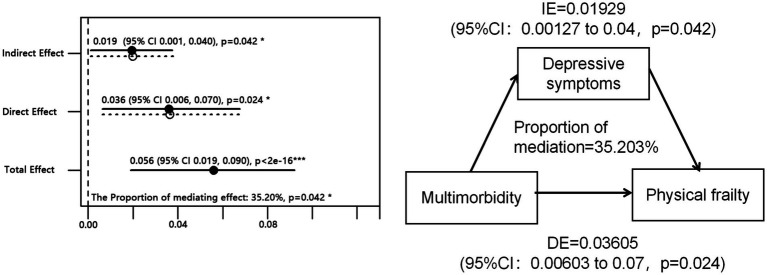
Relationship between multimorbidity, depressive symptoms, and physical frailty.

Then, we investigated the impact of multimorbidity on global frailty and the mediating role of depression. The proportion of the effect of multimorbidity on frailty through depression was found to be 35.2% (*p* = 0.042; see Figure 2, IE = Indirect effect, DE = direct effect). Among middle-aged adults (45 years < age < 60 years), depression accounted for 20.1% of the total effect between multimorbidity and frailty. Conversely, among older adults (age ≥ 60 years), depression mediated 69.8% of the total effect of multimorbidity on frailty, surpassing the direct effect of multimorbidity on frailty (see Supplementary Figure S2).

**Figure 3:**
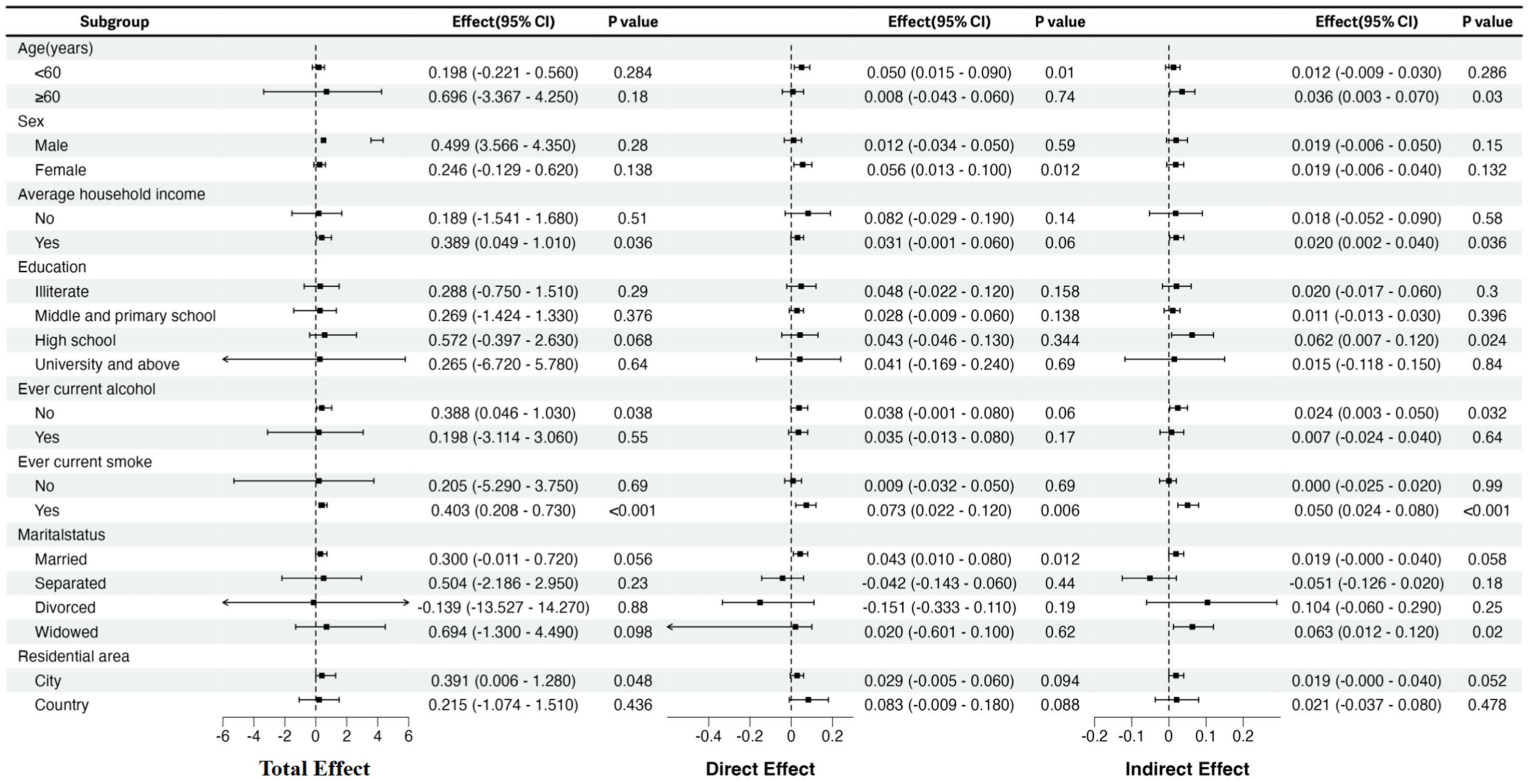
Subgroup analysis: the effect of multimorbidity exposure on physical frailty stratified by age, gender, type of residence, marital status, smoking status, drinking status, average household income and education. CI, confidence interval.

In subgroup analysis, we explored 8 potential effect modifiers (age, gender, type of residence, marital status, smoking status, drinking status, average household income, and education). Figure 3 illustrates that the total effect of multimorbidity is significant among urban residents, smokers, non-drinkers, and individuals receiving economic support. For smokers, depression partially mediates the relationship between multimorbidity and frailty. Among those receiving economic support, individuals with higher education levels and those abstaining from alcohol consumption, the impact of comorbidities is entirely mediated by depression. No significant indirect effects were observed among urban residents.

## Discussion

In this cross-sectional observational study comprising 5,323 adults aged ≥45 years with two or more chronic conditions at baseline, we found distinct multimorbidity patterns to be differentially associated with physical frailty. Compared to the unspecific pattern, all patterns except for ‘memory-related and metabolic disorders’ showed statistically significant associations with frailty in cross-sectional analysis. The magnitude of the odds ratios suggests that the Psychiatric diseases and Cerebrovascular diseases pattern were most strongly associated with physical frailty at baseline, compared with the unspecific pattern. This could simply be an expression of the severity and functional impairment caused by the diseases in these patterns, such as anxiety, depression and cerebrovascular diseases, which have systemic implications (55, 56). Additionally, China has a high prevalence of liver diseases (57), which often lead to coagulation abnormalities, increasing the risk of cerebrovascular diseases (56). Cerebrovascular diseases are frequently associated with respiratory system disorders (58). ‘Cerebrovascular, respiratory, and hepatic diseases’ is characterized by fatigue, prolonged physical inactivity, and metabolic disturbances, which accelerate muscle atrophy and physical decline, thus promoting the development of frailty (59). For cancer patterns, Patients with cancer are also prone to frailty due to the prolonged disease burden and the side effects of treatment (60).

The evidence supporting a bidirectional causal relationship between frailty and multimorbidity continues to grow (2, 21, 47). However, some studies have reported findings inconsistent with ours. For example, a 2019 meta-analysis, comprising nine studies and including data from 14,704 middle-aged and older adult individuals, found that only 868 (6%) were diagnosed with both multimorbidity and frailty (10). This discrepancy in findings may stem from variations in how different studies assess multimorbidity. Despite its widespread adoption and alignment with the deficit accumulation frailty model, operationalizing multimorbidity via disease counts overlooks the intricate nature and interrelationships among illnesses, potentially impacting frailty susceptibility differently (61). Thus, our approach of examining multimorbidity through patterns, rather than relying solely on disease count, offers a nuanced classification and qualitative insight valuable for clinical practice and research (62).

We utilized cluster analysis, an exploratory statistical method, to categorize various multimorbidity patterns based on similarities in disease types and quantities. Recent research has also employed clustering methods, providing a unique opportunity to explore the multimorbidity patterns associated with frailty (63). However, it’s worth noting that our study failed to address the role of psychological factors within these patterns. A recent meta-analysis revealed that more than one-third of older people with physical frailty had depression, and a similar proportion of older people with depression had physical frailty (64). Additionally, a clinical study reported a co-prevalence of frailty and depression at 24.0%, with the incidence of depression in frail subjects reaching 49.4% (39). Furthermore, some studies have suggested that older adults with depression are more prone to frailty than those without depression (65). These findings suggest that depressive symptoms may significantly contribute to the transition from multimorbidity to frailty.

Through mediation analysis, we determined that depressive symptoms can partially influence the relationship between multimorbidity and physical frailty. Although the number of studies investigating the connection between depression-mediated frailty and multimorbidity is limited, the available evidence is notably consistent and supports the findings presented in our study (66). For example, in a longitudinal analysis utilizing data from the Survey of Health, Ageing and Retirement in Europe (SHARE), depressive symptoms observed in Wave 5 data were found to mediate the relationship between frailty identified in Wave 4 data and multimorbidity documented in Wave 6 data among older adult Europeans (67). Similarly, in a cross-sectional analysis conducted by Adame Perez et al., frailty was found to be associated with increased depressive symptoms among patients with diabetes and chronic kidney disease (68). As frailty is multifactorial, it is hypothesized that it serves as a pivotal pathway through which various clinical conditions, such as depression and chronic diseases, lead to a multitude of adverse outcomes (26).

Although this study primarily examined depressive symptoms as a mediator, it is critical to recognize that the association between multimorbidity patterns and frailty may involve multiple pathways. For instance, metabolic syndrome (e.g., insulin resistance, chronic inflammation) could exacerbate frailty by impairing muscle metabolism and energy homeostasis (69). Similarly, inadequate intergenerational family support might reduce access to care resources, indirectly accelerating functional decline (70). Dietary behaviors, such as low protein intake, may directly contribute to sarcopenia and physical frailty (71). While these variables were not measured in the current analysis, future studies should integrate multidimensional mediators—including biomarkers, social support networks, and lifestyle factors—to comprehensively elucidate the mechanisms linking multimorbidity and frailty.

Furthermore, subgroup analyses in our study revealed that age influences the mediating effect of depression, particularly among older adults aged >60 years. This phenomenon has not been reported in other studies to date. The observed age disparity may be attributed to the increasing incidence of chronic diseases among the older people, including hypertension, heart disease, gastric ulcers, arthritis, and diabetes. These individuals often experience prolonged courses of illness and respond poorly to treatment, leading to increasing disability with age. However, focused screening for depression among community-dwelling older adults with these chronic conditions is infrequently practiced, highlighting a critical area for future attention (72).

Based on the study’s findings, it would be crucial to conduct research and community-based rehabilitation management tailored to specific homogeneous groups. Community physicians need to develop rehabilitation programs based on patients’ multimorbidity patterns, incorporating interventions like physical exercise to prevent or delay debilitation. Moreover, there is a need for further research on the biological mechanisms underlying these health outcomes. Particularly, a comprehensive examination of the interplay between multimorbidity, frailty, and depression suggested that routine community screenings should include an assessment of depressive symptoms, in addition to collecting data on risk factors such as chronic diseases and age. Special attention needs to be given to psychosocial changes in older individuals, financial support, smoking, and alcohol consumption, as these factors might lead to psychosocial issues before physical frailty becomes apparent. This perspective outline potential future directions for this study. The temporal sequence of these behavioral patterns and their connection to frailty will be examined in future longitudinal research. Nevertheless, it is crucial to prioritize these factors in older adults within community settings.

## Conclusion

This study presents evidence indicating that distinct multimorbidity patterns have varying associations with the onset of physical decline in middle-aged and older adults in China. Individuals with multimorbidity patterns characterized by psychiatric and cardiovascular diseases may face a higher risk of frailty. Furthermore, the study highlights that depression could serve as a mediator between multimorbidity and frailty, with varying mediating effects across different age groups. Additionally, older adults with financial support, specific multimorbidity patterns, higher education, and no alcohol consumption may be more susceptible to physical frailty mediated through depressive symptoms. Future research should explore the specific mechanisms through which diverse multimorbidity patterns and depressive symptoms contribute to disparities in the risk of physical frailty.

## Data Availability

Publicly available datasets were analyzed in this study. This data can be found at: The datasets generated during and/or analyzed during the current study are available in the CHARLS repository, http://charls.pku.edu.cn.
