# Supplementary figures and images for: The association between multimorbidity patterns and physical frailty among middle-aged and older community-dwelling adults: the mediating role of depressive symptoms
Source: Front Public Health. 2025 May 1;13:1527982. doi: 10.3389/fpubh.2025.1527982 (PMC12078149; doi:10.3389/fpubh.2025.1527982)

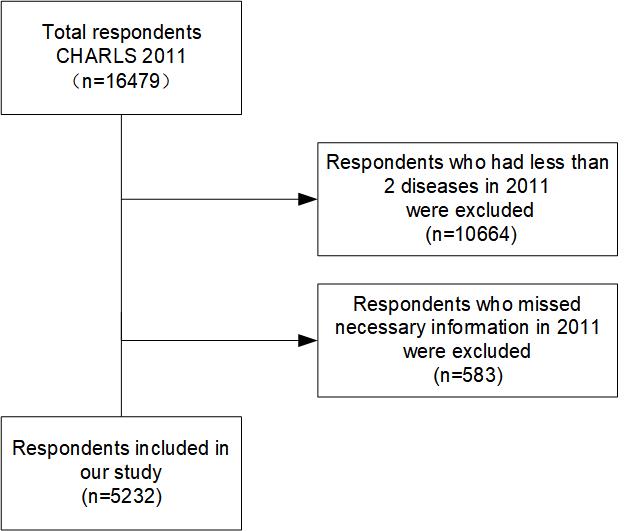

Supplement: Supplementary file 1 [file Image_1.jpeg]

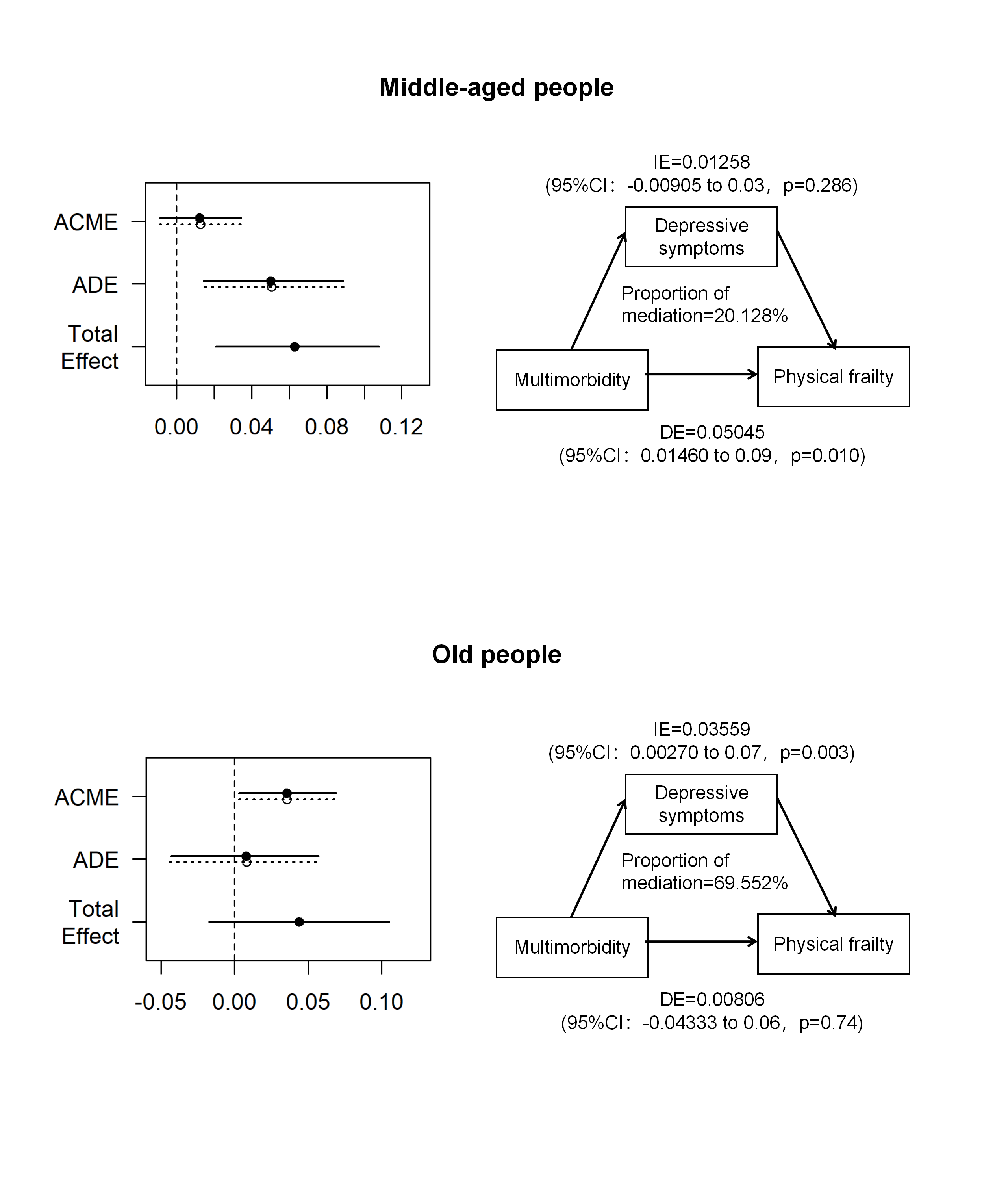

Supplement: Supplementary file 2 [file Image_2.tif]
