# Supplementary material for: The association between multimorbidity patterns and physical frailty among middle-aged and older community-dwelling adults: the mediating role of depressive symptoms
Source: Front Public Health. 2025 May 1;13:1527982. doi: 10.3389/fpubh.2025.1527982 (PMC12078149; doi:10.3389/fpubh.2025.1527982)
Supplement: Supplementary file 3 [file Table_1.docx]

Table S1 Prevalence, observed/expected ratio, and exclusivity of diseases within multimorbidity patterns

| Multimorbidity pattern | Disease | Prevalence within pattern(%) | Observed/Expected Ratio | Exclusivity (%) |
| --- | --- | --- | --- | --- |
| Cerebrovascular disease & Disease of Respiratory system & Hepatic disease | Stroke | 18.7 | 3.51 | 86 |
|  | Asthma | 24.1 | 2.90 | 71 |
|  | Hepatic disease | 20.4 | 2.31 | 57 |
|  | Hypertension | 47.1 | 1.00 | 26 |
|  | Dyslipidemia | 26.0 | 1.21 | 30 |
|  | Heart disease | 39.5 | 1.37 | 34 |
|  | Chronic lung disease | 32.0 | 1.50 | 37 |
|  | Arthritis Rheumatism | 52.6 | 0.86 | 21 |
|  | Kidney disease | 27.6 | 1.91 | 47 |
|  | Digestive system disease | 35.8 | 0.81 | 20 |
|  | Hyperglycaemia | 2.2 | 0.21 | 5 |
| Psychiatric & Cerebrovascular disease | Emotional and mental disorders | 99.2 | 43.3 | 98 |
|  | Stroke | 12.6 | 2.36 | 5 |
|  | Hyperglycaemia | 10.1 | 0.97 | 2 |
|  | Memory Related Disease | 2.5 | 0.90 | 2 |
|  | Hypertension | 29.4 | 0.62 | 1 |
|  | Dyslipidemia | 18.5 | 0.86 | 2 |
|  | Heart disease | 24.4 | 0.85 | 2 |
|  | Chronic lung disease | 16.8 | 0.79 | 2 |
|  | Arthritis Rheumatism | 54.6 | 0.90 | 2 |
|  | Hepatic disease | 10.1 | 1.14 | 3 |
|  | Kidney disease | 14.3 | 0.99 | 2 |
|  | Digestive system disease | 53.8 | 1.22 | 3 |
|  | Asthma | 6.7 | 0.81 | 2 |
| Memory Related Disease & Metabolic | Memory Related Disease | 19.9 | 7.18 | 97 |
|  | Hyperglycaemia | 70.2 | 6.75 | 91 |
|  | Hepatic disease | 17.3 | 1.96 | 26 |
|  | Dyslipidemia | 12.5 | 0.58 | 8 |
|  | Hypertension | 34.9 | 0.74 | 10 |
|  | Heart disease | 23.4 | 0.81 | 11 |
|  | Chronic lung disease | 17.9 | 0.84 | 11 |
|  | Stroke | 3 | 0.56 | 8 |
|  | Arthritis Rheumatism | 52.6 | 0.86 | 12 |
|  | Kidney disease | 18.7 | 1.30 | 17 |
|  | Digestive system disease | 37.9 | 0.86 | 12 |
|  | Asthma | 6.4 | 0.77 | 10 |
| Cancer | Cancer | 100 | 51.29 | 100 |
|  | Hyperglycaemia | 8.8 | 0.85 | 2 |
|  | Memory Related Disease | 2 | 0.72 | 1 |
|  | Hypertension | 37.3 | 0.79 | 2 |
|  | Dyslipidemia | 20.6 | 0.96 | 2 |
|  | Heart disease | 23.5 | 0.81 | 2 |
|  | Chronic lung disease | 13.7 | 0.64 | 1 |
|  | Stroke | 2 | 0.38 | 1 |
|  | Emotional and mental disorders | 2 | 0.87 | 2 |
|  | Arthritis Rheumatism | 58.8 | 0.97 | 2 |
|  | Hepatic disease | 6.9 | 0.78 | 2 |
|  | Kidney disease | 12.7 | 0.88 | 2 |
|  | Digestive system disease | 32.4 | 0.73 | 1 |
|  | Asthma | 3.9 | 0.47 | 1 |
| Unspecific | Digestive system disease | 49.4 | 1.12 | 64 |
|  | Hypertension | 51.1 | 1.08 | 62 |
|  | Dyslipidemia | 21.8 | 1.01 | 59 |
|  | Arthritis Rheumatism | 66.6 | 1.10 | 63 |
|  | Heart disease | 26 | 0.90 | 52 |
|  | Chronic lung disease | 18.1 | 0.85 | 49 |
|  | Kidney disease | 7.9 | 0.55 | 31 |
|  | Hepatic disease | 1.9 | 0.22 | 13 |
|  | Asthma | 2.2 | 0.26 | 15 |

·Shaded cells refer to Observed/Expected ratios > 2 and Exclusivity≥ 25.
