# Supplementary material for: The association between multimorbidity patterns and physical frailty among middle-aged and older community-dwelling adults: the mediating role of depressive symptoms
Source: Front Public Health. 2025 May 1;13:1527982. doi: 10.3389/fpubh.2025.1527982 (PMC12078149; doi:10.3389/fpubh.2025.1527982)
Supplement: Supplementary file 4 [file Table_2.docx]

Table S2 Prevalence of chronic diseases in study population at baseline（n=5232）

| Disease | Number | Prevalence（%） |
| --- | --- | --- |
| Arthritis Rheumatism | 3182 | 61 |
| Hypertension | 2469 | 47 |
| Digestive system disease | 2315 | 44 |
| Heartdisease | 1510 | 29 |
| Chronic lung disease | 1117 | 21 |
| Dyslipidemia | 1124 | 21 |
| Kidney disease | 755 | 14 |
| Hyperglycaemia | 544 | 10 |
| Hepatic disease | 462 | 9 |
| Asthma | 435 | 8 |
| Stroke | 279 | 5 |
| Memory Related Disease | 145 | 3 |
| Cancer | 102 | 2 |
| Emotional and mental disorders | 120 | 2 |
