# Supplementary material for: The association between multimorbidity patterns and physical frailty among middle-aged and older community-dwelling adults: the mediating role of depressive symptoms
Source: Front Public Health. 2025 May 1;13:1527982. doi: 10.3389/fpubh.2025.1527982 (PMC12078149; doi:10.3389/fpubh.2025.1527982)
Supplement: Supplementary file 5 [file Table_3.docx]

The Association Between Multimorbidity patterns and Physical Frailty Among Middle-Aged and Older Community-dwelling Adults: The Mediating Role of Depressive Symptoms

Yuhan Geng, M.D.^¶1,2^, Ming Zhou, Ph.D.^¶1,2^, Yangxiaoxue Liu, M.D.^2^, Tianshu Zhao, M.D.^2^, Jiali Zhang, M.D.^2^, Min Xin, M.D.^2^, Wenxin Wang, M.D.^2^, Gongzi Zhang, Ph.D.^2^^[[1]](#footnote-0)^*, Liping Huang, Prof^2^^[[2]](#footnote-1)^*

^1^Medical School of Chinese PLA, Beijing, China

^2^Department of Rehabilitation Medicine, the First Medical Center of Chinese PLA General Hospital, Beijing, China

**^¶^These authors equally contributed to this work.**

***Correspondence:** Liping Huang and Gongzi Zhang

Supplement Material

Figure S1: Study population derived from CHARLS

Figure S2：Relationship between multimorbidity, depressive symptoms, and physical frailty.

Table S1 Prevalence, observed/expected ratio, and exclusivity of diseases within multimorbidity patterns

Table S2: Prevalence of chronic diseases in study population at baseline（n=5232）

1. [↑](#footnote-ref-0)
2. * Liping Huang Prof, Department of Rehabilitation, Chinese PLA General Hospital, No.28 Fuxing Road, Haidian District, Beijing 100853, People’s Republic of China; huangliping301@163.com, (+86)010-66937857. ORCID:0000-0002-0761-8298

   * Gongzi Zhang Ph.D., Department of Rehabilitation, Chinese PLA General Hospital, No.28 Fuxing Road, Haidian District, Beijing 100853, People’s Republic of China; zgzwoodv@163.com, (+86)010-66935324 [↑](#footnote-ref-1)
